# The effect of metabolic stress on genome stability of a synthetic biology chassis *Escherichia coli* K12 strain

**DOI:** 10.1186/s12934-018-0858-2

**Published:** 2018-01-22

**Authors:** Jillian M. Couto, Anne McGarrity, Julie Russell, William T. Sloan

**Affiliations:** 1 Division of Infrastructure and Environment, School of Engineering, University of Glasgow, Rankine Building, Level 5, Glasgow, G12 8QQ UK; 2 School of Engineering, Infrastructure and Environment, University of Glasgow, Rankine Building, Level 5, Glasgow, G12 8LT UK

**Keywords:** Genome reduction, Chassis, Synthetic biology, Metabolic stress, Mutation rate, Chemostat, Environmental biotechnology, *Escherichia coli*

## Abstract

**Background:**

Synthetic organism-based biotechnologies are increasingly being proposed for environmental applications, such as in situ sensing. Typically, the novel function of these organisms is delivered by compiling genetic fragments in the genome of a chassis organism. To behave predictably, these chassis are designed with reduced genomes that minimize biological complexity. However, in these proposed applications it is expected that even when contained within a device, organisms will be exposed to fluctuating, often stressful, conditions and it is not clear whether their genomes will retain stability.

**Results:**

Here we employed a chemostat design which enabled us to maintained two strains of *E. coli* K12 under sustained starvation stress: first the reduced genome synthetic biology chassis MDS42 and then, the control parent strain MG1655. We estimated mutation rates and utilised them as indicators of an increase in genome instability. We show that within 24 h the spontaneous mutation rate had increased similarly in both strains, destabilizing the genomes. High rates were maintained for the duration of the experiment. Growth rates of a cohort of randomly sampled mutants from both strains were utilized as a proxy for emerging phenotypic, and by association genetic variation. Mutant growth rates were consistently less than rates in non-mutants, an indicator of reduced fitness and the presence of mildly deleterious mutations in both the strains. In addition, the effect of these mutations on the populations as a whole varied by strain.

**Conclusions:**

Overall, this study shows that genome reductions in the MDS42 did not stabilize the chassis under metabolic stress. Over time, this could compromise the effectiveness of synthetic organisms built on chassis in environmental applications.

**Electronic supplementary material:**

The online version of this article (10.1186/s12934-018-0858-2) contains supplementary material, which is available to authorized users.

## Background

Synthetic biology combines advanced molecular and systems biology techniques with principles of engineering design [1, 2]. This field of study is currently designing modified microorganisms, with novel and increasingly sophisticated synthetic biochemical pathways, that have the potential to deliver solutions to some of the world’s most pressing problems in healthcare, energy and the environment [3–5]. For synthetic biology to gain traction it has been necessary to improve and streamline the basic technologies at the heart of the discipline. Thus, there has been a focus on standardization of all of the biological parts in the synthetic biological system. This extends all the way to the genomes of the organisms intended to house the synthetic gene circuits, so reduced or “clean” genome chassis have so far been developed for common lab strains of *Escherichia coli* [6, 7], *Pseudomonas putida* [8, 9] and *Bacillus subtilis* [10].

Scaling-up and placing these engineered organisms in robust biotechnologies is a challenge synthetic biologists are increasingly being forced to confront as the discipline matures [11]. In highly controlled laboratory conditions it has been shown that reduced genome chassis can help with this transition [12, 13] in two respects. First, a reduced genome eases the metabolic burden of both replicating and expressing a large number of genes thus allowing more energy to be directed towards endogenous gene expression [6, 7, 9, 14–16]. Second, by deleting mobile genetic elements, the genome becomes more stable and reproducible, with a reduced likelihood of both mobile-element driven mutagenesis and unwanted byproducts [7, 15]. The benefits of reduced genomes were conceived of with highly controllable white-biotechnologies in mind [12, 13]. However, it remains a moot point as to whether these benefits hold in biotechnologies that routinely experience external perturbations. So, for example, in bioenergy, environmental sensing or resource recovery from waste products it would be naive to expect these engineered organisms to live stress-free. This begs the question; does reducing a genome remain beneficial in the face of, inevitable, environmental stress?

Whilst a myriad of different environmental stressors exist, here we take one by way of an example: starvation. Engineered microorganisms deployed in the environment, in for example, a sensing device would need to ‘live off the land”. Almost all environmental bacterial, almost all of the time, live in oligotrophic conditions; typical carbon concentration of 1–5 mg/L as compared to more-than 2 g/L in typical lab media [17, 18]. These long periods of starvation equate to chronic metabolic stress [18, 19]. In lab studies and in natural microbial populations, starvation causes a decrease in growth rate and an increase in transient mutation rates termed “stress-induced mutagenesis” [20–22]. This phenomenon can promote the emergence of hypermutable subpopulations, which can become temporarily advantageous and contribute to persistence of the entire population [22, 23]. However, for a population of engineered organisms with reduced genomes that are intended to remain stable and perform reliably in a biotechnology, the rapid emergence of mutants introduces an element of unwanted unpredictability, where the resultant loss or gain of function could ultimately compromise the technology.

In this study, the *E. coli* reduced genome strain MDS42 was used as an example of a chassis that was engineered for stability. It was created by systematically deleting non-essential genes, mobile DNA elements and cryptic prophages from the MG1655 strain, while retaining optimal growth properties, and has been described and extensively characterized elsewhere [6, 7, 15, 24]. Its genome is 14.30% smaller than MG1655 and has lost, *inter alia,* all insertion sequence (IS) elements [6]. A fluctuation analysis showed that the spontaneous background mutation rate was 2.4-fold lower in the MDS42 strain than in the parent MG1655 strain, evidence in support of this engineered stability [6]. We questioned whether targeted genome reductions that promote stability would offer an advantage over a non-reduced genome in a stressed environment. Concordant with ‘streamlining theory’ (for example: [25]), a reduced genome increases the efficiency of a microorganism, enabling it to persist in a depleted environment. This ability to persist despite limited resources could translate into reduced instances of stress-induced mutagenesis. In contrast, genome reductions might eliminate compensatory pathways, increasing the overall effect of mutations on the organism’s genome. To test our hypothesis, we grew and maintained populations of both the reduced-genome (MDS42) and their parent strain (MG1655) under sustained metabolic stress while we quantified and compared mutation rates and their effect on fitness and stability.

Triplicate chemostats were utilized, which enabled us to simulate sustained starvation conditions via glucose-limitation [26–28] over a period of 21 days (504 h or 73 generations). Importantly, it is thought that steady state growth, only achievable in chemostats, is closer to the state in which microbes grow in their natural environment [29]. Therefore we grew and maintained steady populations of *E. coli* K12 multiple deletion series (MDS) strain MDS42 [6]. Then, in identical conditions, we grew and maintained steady populations of the parent K12 MG1655 strain, which served as a control. We quantified spontaneous mutation accumulation in both sets of populations periodically. We show that within 24 h both strains accumulated mutations at a similar rate. Furthermore, these mutation rates were dynamic in strains from both populations and increased overall, showing that this engineered stability was insufficient when strains underwent metabolic stress. Maximum growth rates of mutants isolated from both cultures were consistently less than those of the non-mutants suggesting that the mutations were mildly deleterious in both strains, however their effect in each population was different. High rates of emerging genetic variation introduce a level of unpredictability that could ultimately compromise the function of engineered organisms and adversely affect the biotechnology they are embedded in.

## Results

**Fig. 1:**
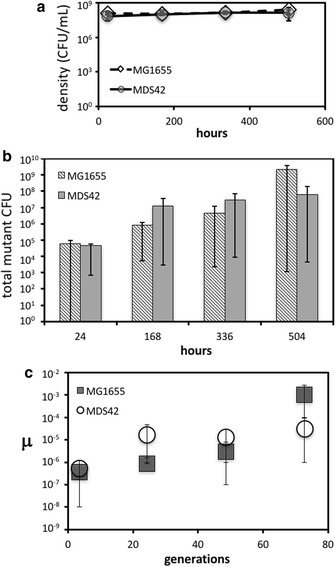
A description of mean steady-state growth, mutant accumulation, and mutation rate for all chemostat runs: n = 3 per strain. The vertical scale is logarithmic (Log_10_) and the horizontal scale is in hours, for all three sections of this figure. **a** The relationship between mean cell density measured as colony forming units per milliliter (CFU/mL) and time measured in hours. Cell density for all runs reached steady state in approximately 20 h and stayed that way till the end of the sampling period. The diamond shape and dotted line represents the mean cell density for the MG1655 strain. The circle and the solid line represent mean cell density for the MDS42 strain. Horizontal error bars represent the standard deviation above and below the mean. **b** The relationship between the mean of the total number of mutant colony forming units (*cyc*^R^, *rif*^R^) for each chemostat run and time (in hours). The diagonally stripped boxes represent the means for the runs containing the MG1655 strain and the solid grey boxes represent the means for runs containing the MDS42 strain. Standard deviations are not shown, as some were negative values, which cannot be plotted on graphs that utilise a logarithmic scale. Instead the horizontal error bars represent the distance between each mean and the maximum and minimum number of mutant colonies quantified at each time point. **c** The mean mutation rate (μ) measured as mutations per cell per generation, that was estimated at four time points (24, 168, 336, 504 h) during the experiment. The horizontal axis is scaled to generations, where the generation rate was 0.14 generations per hour for a growth rate (η) of 0.1 hr^−1^. The grey squares represent the MG1655 strains and the open circles represent the MDS42 strains. The horizontal error bars represent the standard deviation above and below the mean mutation rate

Growth rates (denoted as ‘η’ in the current study), and hence generation times, of both the MG1655 and the MDS42 strains have been previously compared and have been shown to be unequal [24]. We confirmed these findings via batch growth in the minimal glucose-depleted medium that was utilized in all experiments in our study. Doubling times were 38 (1.1 h^−1^) min for the MG1655 strain and 53 (0.79 h^−1^) min for the MDS42 strain (Additional file 1: Figure S1). Unequal generation times have a bearing on mutation rates thus utilizing a chemostat environment enabled us to fix the growth rate (η) and generation time [26, 30] in both strains, allowing for a direct comparison. In addition, controlling dilution rates and the carbon source (substrate) concentrations can allow for bacterial growth under a constant selective pressure, which in this experiment was hunger and starvation [26–28]. Triplicate populations (N = 3) of both strains of *E. coli* were grown in continuous culture in the chemostats that were fed a substrate of minimal glucose-depleted media. A dilution rate of 0.1 h^−1^ ensured that the substrate in the chemostat was depleted and yielded a generation time of 6.9 h. Previous work with derivatives of the *E. coli* K12 strain that have not been engineered to remain stable reported that they acquire mutations readily in glucose-limited chemostats [20]. Therefore, it was expected that mutations would accumulate in the triplicate MG1655 chemostats, and hence they were utilized here as controls. Populations of both strains reached a steady state within 20 h, maintaining an average density of 1.6·10^8^ colony forming units (CFU) per mL for the MG1655 strain and 1.1·10^8^ CFU*mL^−1^ for the MDS42 strain, with no significant difference between the two (P = 0.128; Fig. 1a).

Spontaneously occurring mutations are extremely rare and are typically observed when they produce a detectable phenotype, usually observed via a selective screen. Fortuitously in *E. coli*, spontaneous mutations produce a detectable phenotype change when they occur at either the RNA polymerase subunit (*rpoB*) locus or the cycloserine A (*cycA*) locus. Specifically, mutations at the *rpoB* locus render an organism resistant to the antibiotic Rifampicin (*rif*^R^) [31] and mutations at the *cycA* locus confer resistance to d-cycloserine (*cyc*^R^) [32]. Therefore antibiotic-containing selector plates can be used to screen for these specific mutants. Although mutations at different loci and/or silent mutations are excluded here, this is a commonly used system with the *cycA* locus especially being used to estimate background mutation rates in *E. coli* (see Ref. [32] for example). Indeed the *cycA* locus was used for the first estimate of background mutation rates in the MDS strains [6]. Here we exploited this system by sampling the continuous culture populations periodically and plating these samples on selector plates to quantify spontaneous mutant accumulation over time. Samples were also plated on non-selector plates for a colony count. *Rif*^R^ and *cyc*^R^ mutants accumulated in both the MG1655 and MDS42 strains, with marked variation between runs (Fig. 1b). Mutation rates (denoted as ‘μ’ in the current study) were estimated using a simple linear mutation accumulation model, that reports the number of mutations per cell per generation and is suitable for chemostats [33] (Table 1; Fig. 1c). Mutation rate estimates (μ) in the MDS42 strain were 1.4-fold higher than in MG1655 by 24 h, increasing to approximately 20-fold higher by 168 h (7 days) (Table 1). These were then surpassed by the MG1655 strain 504 h into the experiment, where the average estimated mutation rate reached 1.03·10^−3^ mutations cell^−1^ generation^−1^, which is approximately 30-fold higher than in the MDS42 strain (Table 1; Fig. 1c).

**Table 1:** Estimates of mutation rates (μ) in mutations/cell/generation for the MG1655 and MDS42 strains

*MDS42*	*24* (3.5)	*168* (24.2)	*336* (48.5)	*504* (72.7)
*cyc* ^*R*^	2.99·10^−7^	7.07·10^−7^	2.27·10^−5^	3.04·10^−5^
*rif* ^*R*^	2.31·10^−7^	1.67·10^−5^	− 9.21·10^−6^*	1.51·10^−6^
Total	5.30·10^−7^	1.74·10^−5^	1.35·10^−5^	3.19·10^−5^
*MG1655*	*24* (3.5)	*168* (24.2)	*336* (48.5)	*504* (72.7)
*cyc* ^*R*^	1.03·10^−7^	6.30·10^−7^	2.97·10^−6^	4.34·10^−4^
*rif* ^*R*^	2.72·10^−7^	2.36·10^−7^	− 6.52·10^−8^*	5.99·10^−4^
Total	3.76·10^−7^	8.66·10^−7^	2.91·10^−6^	1.03·10^−3^
Fold diff. MDS42:MG1655	1.41	20.05	4.63	0.03

Mutation rates for each strain (italics) were estimated separately at each locus, and then as a total. The time at which each sample was quantified is indicated in hours (italics) at the top of each column, with the number of generations, a scaled measure of time, indicated in the brackets below. The term ‘fold diff.’ refers to the ratio of the total mutation rate for MDS42 to MG1655 (μ_MDS42_/μ_MG1655_). The asterisks mark two negative mutation rate estimates. This occurred because *rif*^*R*^ mutant colony counts for each strain during one out of three chemostat runs were slightly lower at 336 h compared to at 168 h, which produced a negative value

The spontaneous mutation rate in both strains had previously been estimated at the *cycA* locus via a fluctuation test [6]. Working under the assumption that these were an estimate of a background mutation rate, we compared the rates acquired in our chemostats at the *cycA* locus only (Table 1), with these previous estimates, and found an approximately eight-fold increase in just 24 h in the MDS42 strain and a two-fold increase in the MG1655 strain (Additional file 1: Figure S2). This fold-difference increased further in both strains in comparison with this background rate estimate.

**Fig. 2:**
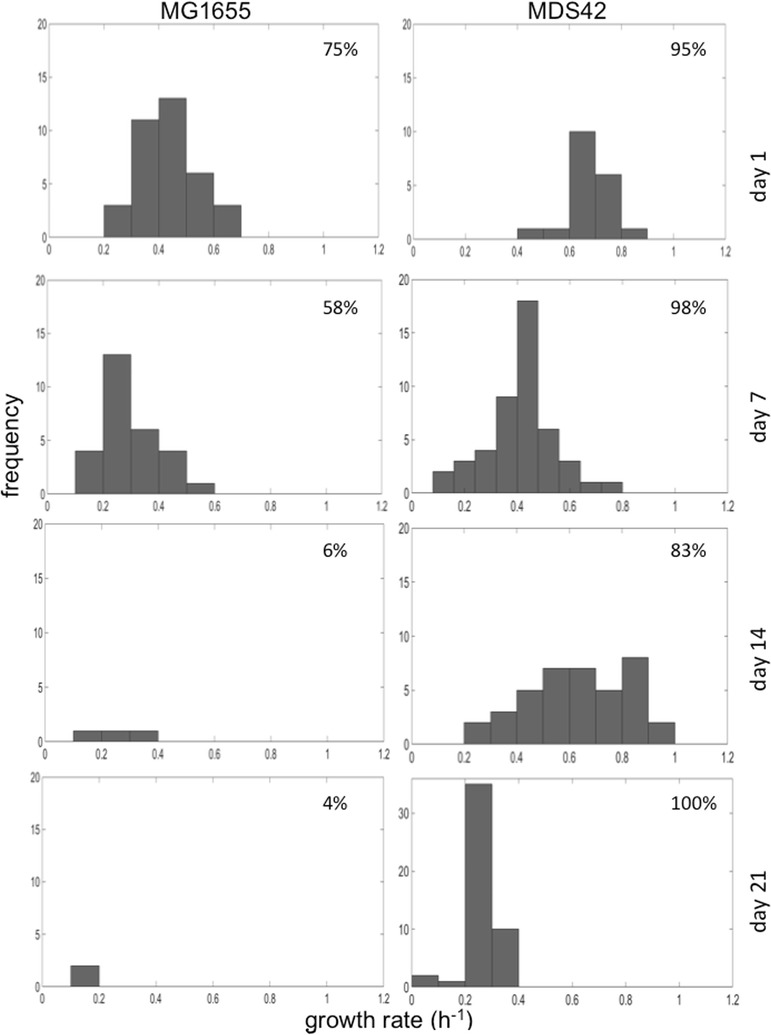
The distribution of individual growth rates, ascertained via batch culture, for the isolated *cyc*^R^ mutants sampled from intervals starting at 24 h (day 1), and then seven, 14, and 21 days respectively. Forty-eight mutants were isolated from each time point from each strain except for the 24-h interval for MDS42, where only 20 mutants were available. Each mutant was grown individually in batch culture using the same growth medium as used in the chemostat cultures. The percentage of mutants that had a growth rate that was greater than zero is indicated in the top-right corner of each panel

**Fig. 3:**
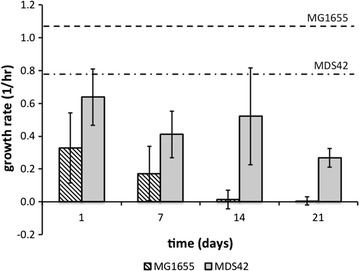
A comparison of mean *cyc*^R^ mutant growth rates of batch monocultures of the MG1655 (stripped bars) and MDS42 strains (grey bars). The two horizontal lines represent the mean growth rate for non-mutant strains of MG1655 (dashed line; 1.1 hr^−1^) and MDS42 (dotted-dashed line; 0.79 hr^−1^)

In a chemostat, growth rate is an important measure of fitness. The mean growth rate is held constant by controlling the dilution rate. This means that if any portion of the population gains an advantage it is at the expense of the remainder. So, faster growing mutants will ultimately dominate and slower growers will ultimately wash out of the population. Thus, growth rate was used as an index to phenotypic variation. We reasoned that in a clonal population, a wider distribution of growth rates was indicative of increased phenotypic, and hence, by association, emerging genetic variation. Previously published work [34, 35] has shown that phenotype screening is an effective way of assessing the emergence of genetic diversity in chemostat populations. Therefore, populations of mutants from two chemostat runs—one for each strain—where the mutant fraction increased monotonically over time were chosen for further investigation. Forty-eight *cyc*^R^ mutant colonies were chosen at random from each time point with the exception of the MDS42 mutants sampled at 24 h; a total of 20 colonies was available here. Each mutant was grown individually in batch culture using the same minimal glucose-depleted medium as used in the chemostat experiments. Twenty-four non-mutants from both strains were also grown in the same conditions for comparison. These non-mutant populations were streaked from fresh glycerol stocks and were thus assumed to have no mutations. To check that tradeoffs between growth and yield did not confound our assertion that growth rate is a reasonable measure of fitness, we estimated biomass yield in the batch experiments and showed that the average yield of mutant strains was lower (MG1655) or the same as (MDS42) the non-mutant strains (Additional file 1: Figure S3). The observed growth rate distributions were different between strains (Fig. 2). Of the MDS42 mutants, 83–100% sampled through time were able to grow in isolation in batch. The overall mean mutant growth rate for the MDS42 mutants was 1.2-fold slower than non-mutant growth under the same conditions in batch (Fig. 3). Seven days into the experiment, we observed a wide range of growth rates, which expanded further by day 14 (Fig. 2); a small percentage of mutant growth rates also approached the mean rate of non-mutant growth (Fig. 3). Following this, the distribution of growths for mutants sampled on day 21 had a smaller range and a lower mean (Fig. 2).

**Fig. 4:**
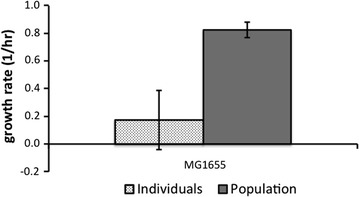
Analyses of the cross feeding (CF) phenotype that was detected amongst the MG1655 *cyc*^R^ mutants. Mean growth rates of 48 *cyc*^R^ mutants that were grown in batch monocultures (dotted) and as a group (grey)

In comparison, 4–75% of the MG1655 mutants grew in isolation. For the MG1655 mutants sampled we observed a similar broad range of growths after day 7 (Fig. 2). Here too mean mutant growth rates were much slower than the non-mutant populations (Fig. 3). The rate of decline in the mean and variance of growth rate of mutants in batch was far greater in the MG1655 strain. By day 14 only three of the mutants grew in pure batch culture, which dropped to only two by day 21 (Fig. 2). This was unexpected because the mutant fraction in samples from the chemostat for this particular experimental run was very high (5.03% by day 21). We questioned whether this observation was the result of individuals in the population with cross-feeding polymorphisms. Cross-feeders are individuals that only grow well as part of a group and have been known to emerge in glucose-limited chemostats [34]. If this were the case then reconstituting the mutant population would change the mean growth rate. We tested this with one of the mutant populations and found that when we mixed the mutants, we observed a 4.8-fold increase in growth rate compared to when each was grown individually (Fig. 4).

The observation of a relative increase in mutant growth rate, when grouped, led us to investigate whether this was a general phenomenon that occurred in all the chemostats. A chemostat environment ensures that cell density is controlled by the dilution rate (steady state). Although cell density remained fairly stable over time (Fig. 1a), there were still some fluctuations observed that were quantified as deviations from the mean. An increase in the growth rate in a portion of the population would be expected to yield a temporary rise in density until the system equilibrates back to the original density and mean growth rate. We tested whether this fluctuation from ‘steady state’ was related to mutant accumulation (mutant CFU/mL) using Kendell’s tau (τ). We found a strong trend towards a correlation (τ = 1.0000; P = 0.0833) between mutant CFU and the variation in cell density of the MG1655 population, but not the MDS42 population (τ = 0.3333, P = 0.7500).

## Discussion

A major motivation for adopting minimal genome chassis organisms in applications of synthetic biology centers on minimising mutations and genetic diversity and thus enhancing stability and reproducibility. These are desirable properties in biotechnologies and there is promising lab-scale evidence [6, 9, 15, 16] that minimal chassis can deliver them for white-biotechnologies, where the environment can be tightly controlled [12, 13]. However, for many proposed applications of synthetic biology, such as in environmental sensors, organisms will be exposed to environmental stress, such as metabolic stress. Evidence from both lab and natural bacterial populations show that stresses increase mutation rates [21–23]. An increase in stress-induced mutagenesis in an engineered organism would have potentially detrimental effects on the organism’s stability and persistence.

Our results show that even in as little as 24 h of metabolic stress, the MDS42 strain accumulated mutations just as quickly as MG1655, a strain that was not engineered for genetic stability. Despite marked variation within the runs, the estimated mutation rates rose in both strains suggesting that minimising a genome does not offer the benefits of stability during prolonged metabolic stress. Moreover, the prevalence of slow growth rates for mutants from both strains at all time points were indicative of a drop in fitness. The average biomass yield for both MDS42 and MG1655 mutants were either similar to or lower than yields for non-mutants (Additional file 1: Figure S3) suggesting mutants were unlikely to be adaptive. For the MG1655 mutants we also showed that whilst mixed cultures of mutants grew faster than monocultures their cumulative growth rate did not exceed rates observed in non-mutants. Therefore, taken together the data suggest that the accumulation of mutants is more likely attributable to a continued supply of slow-growing new mutants rather than a few fast growers. Slow growing mutants are more likely to be washed out of the chemostat and therefore our samples are likely to underestimate mutation accumulation. So, the true rate of mutation in our stressed populations is expected to be even higher. Indeed, to demonstrate just how conservative our estimate of mutation rate is we considered the probability that the average mutant is washed out of the chemostat. Suppose that mutants randomly appear and then grow at a rate η_*m*_. Letting *N*(*t*) be the number of individuals of a particular mutant, *t* hours after they first appeared. Then assuming *N*(*t*) is continuous,

1 $$\frac{dN(t)}{dt} = \eta_{m} N(t) - \frac{Q}{V}N(t),$$

where *Q* is the flowrate into and out of the chemostat and *V* is the volume and hence, *Q*/*V* is the dilution rate, which fixes the mean growth rate. So, replacing *Q*/*V* with *η* the growth rate of the non-mutant majority and solving we get

2 $$N(t) = N_{0} \text{e}^{{(\eta_{m} - \eta )t}} ,$$

where *N*_0_ = *N*(*t* = 0). Given that in our case *N*_0_ = 1 if we assume that births and loss occur randomly in the period *t* we can approximate the probability, *P*, that the mutant population has left by *P* = 1 − *N*(*t*) and so,

3 $$1 - P = \text{e}^{{(\eta_{m} - \eta )t}} .$$

Therefore, we can estimate the time taken for the probability of washout of the mutant subpopulation to be *P* as

4 $$t = \frac{\ln (1 - P)}{{(\eta_{m} - \eta )}}$$

In our case the dilution rate and hence growth rate (η) is 0.1 h^−1^. We assume that the mutant, with lower growth rate, does not become abundant enough to affect the overall population growth rate and hence η remains constant. The average mutant in the MDS42 strain grows half as fast as a non-mutant in the chemostat and therefore, η_*m*_ = 0.05 h^−1^. So with probability, *P* = 0.99, the mutants will have washed out in t = 92.1 h. Thus, when mutants grow half as quickly as the general population, then we can be 99% confident that those that we see in a sample appeared in the population within the past 92 h (Additional file 1: Figure S4 for the full distribution). So in our case when we see 6.52 × 10^7^ mutants (the average of the three MDS42 chemostat runs at 21 days) in the population, a mutant has appeared on average every 0.005 s.

It is generally thought that new mutations are usually neutral or deleterious and beneficial mutations are very rare ([36]). Moreover if the mutation has occurred in a gene that only makes a small contribution to a particular phenotype, the mutation might appear ‘silent’, with little or no discernible effect over the (non-mutation driven) major transcriptional changes that make large contributions to the overall phenotype under investigation. Indeed, metabolic stress and fluctuating reactor conditions elicit transcriptional changes in chemostat populations of *E coli* in a matter of seconds, leading to a new ‘steady state’ that is presumably reached by all cells within these populations [37, 38]. It is reasonable to assume that this has occurred in both the MDS42 and MG1655 chemostat populations in the present study. However, our screen enabled us to select individual mutants from these populations, and their growth rate phenotypes varied, suggesting that we were able to observe additional changes to this phenotype that deviates over and above the new steady state. Although our growth rate distributions (Fig. 2) suggested a high turnover of phenotypes, brought on by an elevated mutation rate, the fitness effect of mutations, and whether they could lead to adaptation were not clear. For the MDS42 mutants the first 14 days saw a wide distribution of slow growth rates, an indicator that mutations were likely to be deleterious, but insufficiently so to see them washed out of the chemostats quickly. Beyond 14 days a smaller range of growth rates was observed, which could be indicative of either a decrease in the accumulation of new mutants, or the possibility that the sustained high mutation rates led to the acquisition of increasingly deleterious phenotypes. This is plausible given that IS elements have been deleted from the MDS42 strains. Previous reports have suggested that the systematic deletion of IS elements hinders the ability of these multiple deletion strains to evolve in that acquired mutations, even with a detrimental effect on growth, are not fatal to the cell [15, 24]. Therefore, even in instances where high mutation rates are sustained, new mutations of varying effects continue to accumulate rapidly in MDS42 populations, which over time could make the population more susceptible to genetic drift or purifying selection events.

In contrast, the range and mean of the distribution of mutant growth rates in the MG1655 strain appear to fall after just 7 days in batch monocultures. In addition, by day 14, 94% of mutants would not grow in monocultures and those that did, grew very slowly, an indication of deleterious mutations. Yet surprisingly, mutant accumulation in the chemostats continued to rise over the 21 days. Furthermore, in mixed batch cultures, mutants appeared to thrive, with a growth rate approximately four times faster than when grown individually. So it appears that for the strain with no genome reductions, MG1655, the mutants that survive in the chemostat are no longer exploiting the same niche as the non-mutants; they do not grow in the original media. Their ability to thrive as a cohort suggests that in these strains, acquired mutations have delivered cross-feeding phenotypes. Cross-feeders metabolise by-products from other individuals in the population [39, 40]. Therefore, in a chemostat, a limited but renewed source of glucose could establish a hierarchy whereby a percentage of the population utilises this glucose, and produces byproducts that could, in turn, feed other members of the population, allowing cross feeders to adapt. Multiple byproducts could open up a multitude of niches, leading to the adaptation of several putative beneficial mutations. This could explain the weak, but present, correlation between mutant accumulation and deviation from steady state in the MG1655 chemostats, which suggests accumulating mutations were causing an increase in cell density. This correlation was absent in the MDS42 reactors.

Overall, our study showed that stress increased mutation rates in both reduced and non-reduced *E. coli* strains. The MDS42 strain has been engineered for stability and was intended for a closed and controlled biotechnological application [6]. Although the current study shows that the elevated mutation rate was the result of metabolic stress that is typical of an open environment, it stands to reason that closed systems would behave similarly when under any stress. Previously documented evidence from lab evolution studies using *E. coli* show that similar to any other stress, glucose-limitation in a chemostat elicits the general stress response affecting genes such as *rpoS,* a master regulator of this stress response [27]. This stress-response can also down-regulate the DNA mismatch-repair machinery via mutations in *mutS* and *mutY* which ultimately leads to an increase in background mutation rates [21, 22, 26]. Although not specifically assayed in the current study, these mutations are very likely to have occurred in our populations too. Moreover, chromosome replication times have been observed to change in stressed chemostat populations in association with altered expression of genes involved in DNA replication, repair, and recombination [41]. Here too, these genes are triggered by and control a multitude of processes involving DNA integrity and hence could lead to an increase in mutation rates if mis-expressed.

Given that stress can lead to mutations via so many different genetic pathways, it is reasonable to assume that a stress of any type, even in a closed system could lead to an elevated mutation rate. This study also found that the adaptive effect of mutations was difficult to predict. We observed a different effect in populations from each strain, neither of which would be ideal for a biotechnology. For example, the cross-feeding mutants that emerged in the MG1655 populations could produce unwanted byproducts. Moreover, if embedded in an environmental biotechnology, the adaptations could lead to the engineered microorganisms becoming bio contaminants. In the MDS42 strain, the deletions of IS elements appeared to initially ‘dampen’ the effect of mutations a potential advantage for a biotechnology, provided that the process was completed in a few days. For longer-term applications, or for proposed environmental biotechnologies, further chassis modifications would be required.

## Methods

### Bacterial strains

Strains of bacteria used in this study are both derivatives of *E. coli* K12. The MG1655 (F- lambda- *ilvG*- *rfb*-50, *rph*-1) substrain is the *E. coli* reference strain (GI: 556503834) and was purchased from the DSMZ culture collection (Braunsweig, Germany). The multiple deletion series (MDS) MDS42 substrain (GI: 471332236) was created by deleting IS elements, cryptic prophages, and degenerate genes from MG1655 and has been described in detail in Posfai et al. [6]. It was purchased from *Scarab Genomics* (Wisconsin, USA).

### Chemostats and culture conditions

Strains were streaked onto fresh LB-agar plates from glycerol stocks and growth overnight at 37 °C. A single colony was then inoculated into a 100 mL culture of freshly prepared minimal M9 media [42] supplemented with 10 mM glucose and 0.2% casamino acids and grown till it reached an optical density of 0.1. Eight millilitres of this culture was used to inoculate a 500 mL computer controlled-chemostat (modified miniBio^®^ 500, Applikon Biotechnology, Delft, NL). The continuous culture was maintained at a volume of 350 mL with constant stirring at a dilution rate of 0.1 hr^−1^ at a temperature of 37 °C and a pH of 7.00 for 504 h (21 days). Chemostat cultures were maintained in a glucose-depleted medium, which was made by supplementing a minimal M9 medium [42], with 1 mM glucose and 0.2% casamino acids. Amino acids were utilised as a result of repeat test growth experiments with both strains in batch culture (data not shown). We found that growth for both strains was more consistent across tests when the medium was supplemented with amino acids. The optical density was monitored both continuously using the bugLab^®^ BE 2100 biomass monitor (Applikon Biotechnology, Delft, NL) and periodically, every 24 h, via subsampling and measurement on the Infinite^®^ m200 Pro automated microplate reader (Tecan Group Ltd., Männedorf, Switzerland). In these conditions, the OD600 reached 0.1 in about 20 h (steady state) and remained that way for the duration of the experiment.

### Mutant fraction and mutation rate estimates

At each sampling period (24, 168, 336, and, 504 h) ~ 2–5 mL of the reactor liquor was subsampled using sterile conditions and techniques. Of this volume, 1 mL was utilised to make a glycerol stock, while 1 mL each was plated onto 5 LB-d-cycloserine (100 μg/mL) plates and 5 LB-Rifampicin (50 μg/mL) plates. All plates were freshly prepared. An additional 1 mL was diluted and plated onto LB-agar plates for a colony count. All plates were incubated overnight at 37 °C. Spontaneous mutations occurred and accumulated in populations from both strains and were scored via acquired resistance to either d-Cycloserine (*cyc*^R^) or Rifampicin (*rif*^R^). Mutation rate (μ) was estimated using a mutation accumulation model [33],

5 $$\mu = \left( {\frac{1}{N\lambda }} \right)\left( {\frac{{r(t_{2} ) - r(t_{1} )}}{{t_{2} - t_{1} }}} \right),$$

where *N* is the constant cell number in a chemostat, and *r(t)* is the number of mutations measured at time (t) and t_1_ < t_2_ are two discrete time points. The term λ is generations per unit time (generations hr^−1^) [43], where

6 $$\lambda = \frac{\eta }{\ln (2)},$$

and η is the growth rate in the chemostat of 0.1 hr^−1;^ therefore, λ equals 0.14 generations hr^−1^. Time, in ‘hours,’ is eliminated from Eq. (5), leaving generations, which is a scaled measure of time commonly used in genetics to report mutation rates. Therefore using Eq. (5), mutation rate (μ) is expressed as mutations per cell per generation (mutations cell^−1^ generation^−1^).

### Mutant monoculture batch growth conditions

Up to 48 randomly picked colonies were batch cultured individually at 37 °C overnight in minimal media supplemented with 10 mM glucose and 0.2% casamino acids. Each culture was then diluted 100-fold into fresh M9 supplemented with 1 mM glucose and 0.2% casamino acids. Cultures were then grown (in batch mode) at 37 °C and optical density at 600 nanometres (OD_600_) was measured every 20 min in the Infinite^®^ m200 Pro microplate reader (Tecan Group Ltd., Männedorf, Switzerland) for a period of 17 h. Blank measurements were also obtained and subtracted from OD_600_ at each time point. Growth rate (h^−1^) was estimated as the slope of the straight-line portion of the plot of the natural log of each blank-corrected OD_600_ measurement (Ln[OD_t_-blank]) versus time. Yield was equivalent to the final OD600 measurement at the end (17 h) of each batch culture experiment.

## Additional file

**Additional file 1: Figure S1.** Mean growth rates of 24 individual colonies each of the MG1655, and the MDS42 strains from batch monocultures. **Figure S2.** Fold-difference between mutation rates estimated from the current study (stress) and Posfai et al. (2006). **Figure S3.** Average biomass yield for mutants and non-mutants of the MG1655 and MDS42 strains. **Figure S4.** Wash out time distributions for isolated cyc^R^ mutants from both the MG1655 and MDS42 strains at different time points during the experiments.

## Abbreviations

CFU: colony forming units
OD: optical density
LB: Luria-Bertani broth
IS: insertion sequence
MDS: multiple deletion series
*cycA*: cycloserine A
*rpoB*: RNA polymerase β subunit
rif^R^: Rifampicin resistant
cyc^R^: d-cycloserine resistant
