# Supplementary material for: The effect of metabolic stress on genome stability of a synthetic biology chassis Escherichia coli K12 strain
Source: Microb Cell Fact. 2018 Jan 22;17:8. doi: 10.1186/s12934-018-0858-2 (PMC5776760; doi:10.1186/s12934-018-0858-2)
Supplement: Supplementary file 1 — Additional file 1: Figure S1. Mean growth rates of 24 individual colonies each of the MG1655, and the MDS42 strains from batch monocultures. Figure S2. Fold-difference between mutation rates estimated from the current study (stress) and Posfai et al. (2006). Figure S3. Average biomass yield for mutants and non-mutants of the MG1655 and MDS42 strains. Figure S4. Wash out time distributions for isolated cycR mutants from both the MG1655 and MDS42 strains at different time points during the experiments. [file 12934_2018_858_MOESM1_ESM.docx]

**Additional File 1:**

Jillian M. Couto^1*^, Anne McGarrity^1^, Julie Russell^1^, William T. Sloan^1^

1: Division of Infrastructure and Environment, School of Engineering; University of Glasgow, Glasgow G12 8QQ

This document contains four figures, S1- S4 that complement the main text.


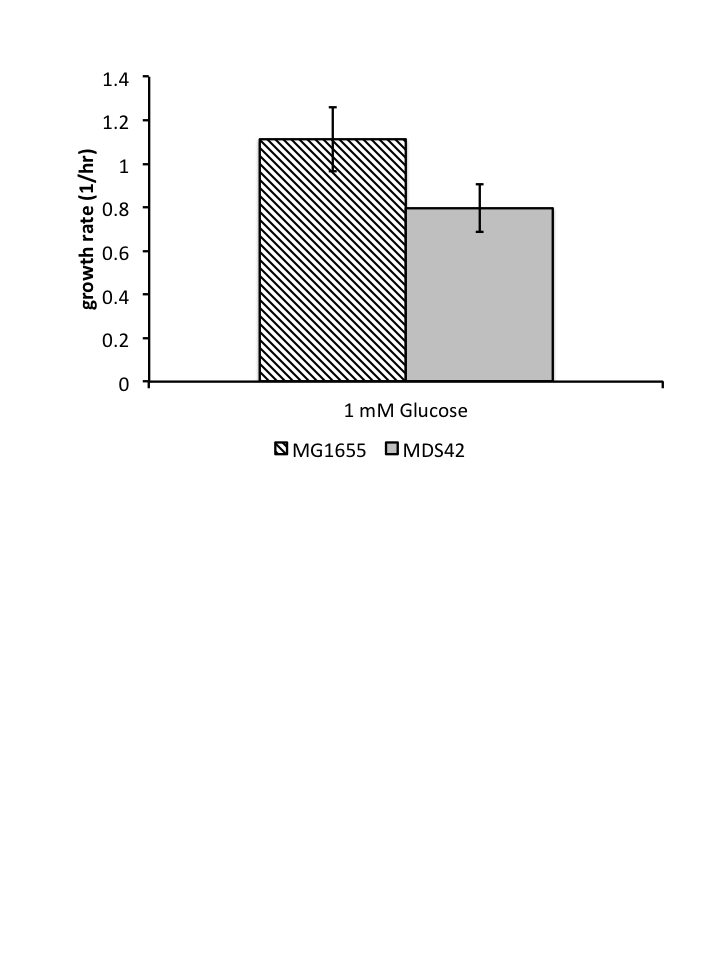


**Figure S1:** Mean growth rates of 24 individual colonies each of the MG1655 (stripped bar) and the MDS42 (grey bar) strains in batch monocultures.


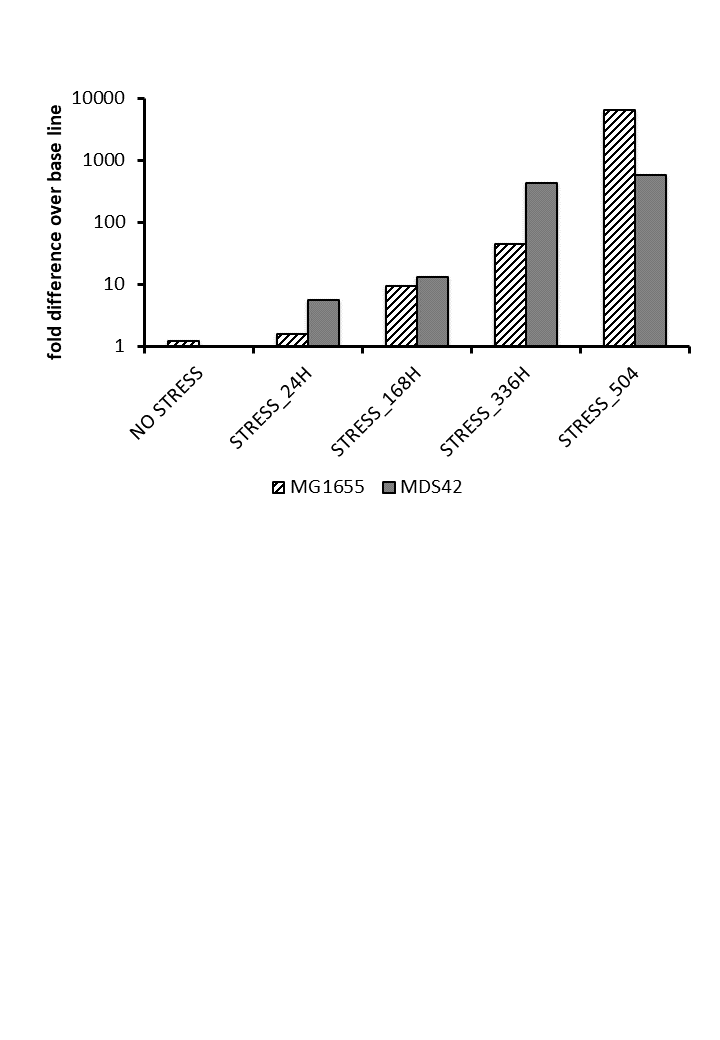


**Figure S2:** Fold-difference between mutation rates estimated from the current study (stress) and Posfai et al (2006), where background mutation rates were estimated over a 24-hour period via a LB-fluctuation test.


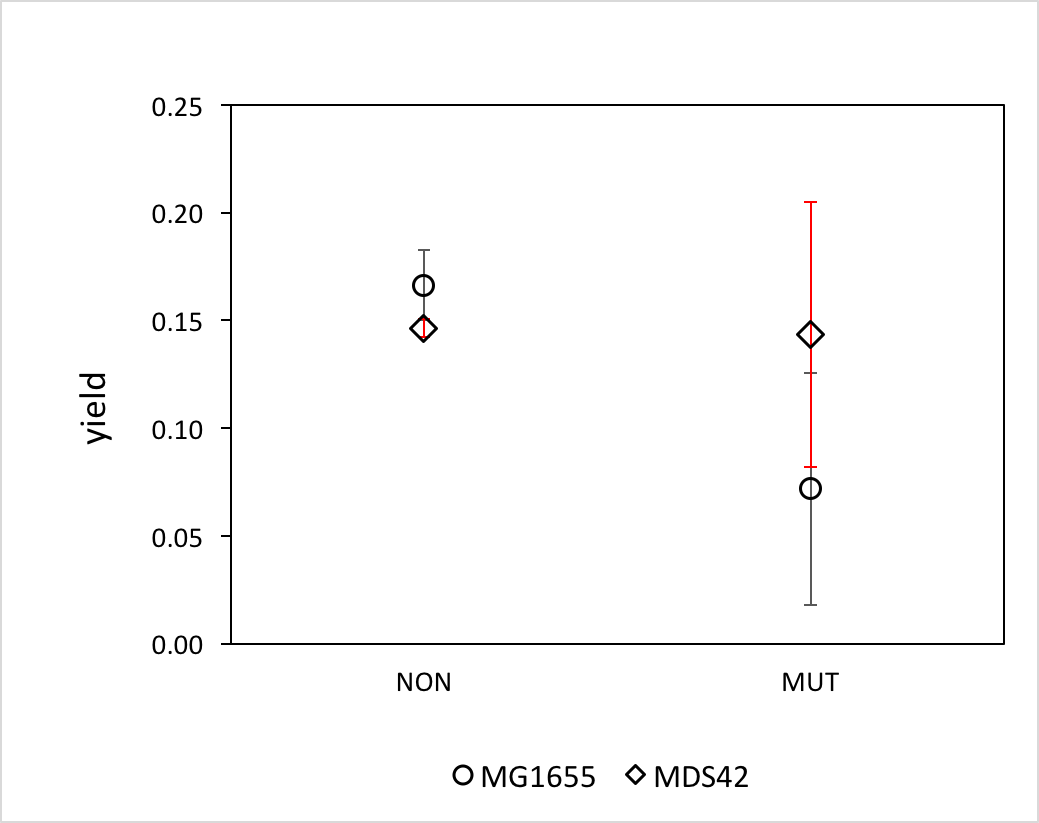


**Figure S3:** Average biomass yield for non-mutants and mutants of both the MG1655 (circle) and MDS42 (diamond) strains. In this study, yield was equivalent to the final OD_600_ measurement which was taken at the end of each batch culture experiment, which lasted for 17 hours. The yield for each mutant that produced a non-zero growth rate (Figure 2) from all four time points was averaged for each strain. Twenty-four non-mutants were also grown in batch culture as a comparison (Figure S1). Yield values for these were also averaged. Error bars (MDS42 = red and MG1655 = black) represent the standard deviation above (plus) and below (minus) the average for each strain.


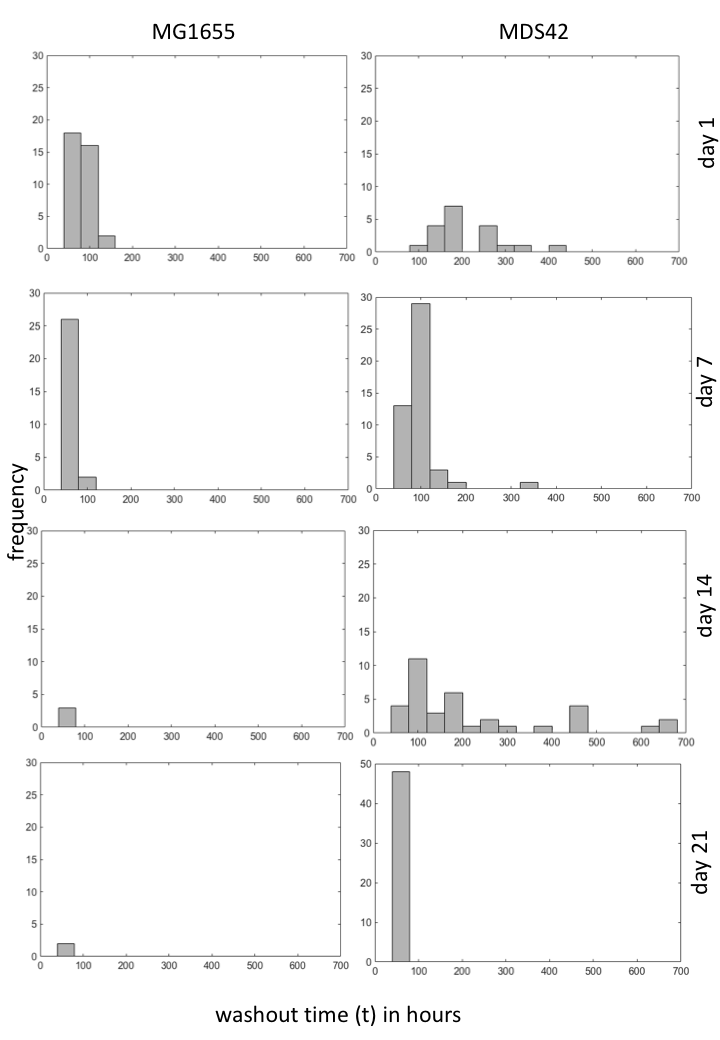


**Figure S4:** Distribution of washout time measured in hours for each cyc^R^ mutant that was isolated from the chemostats at 24 hours, and then seven, 14, and 21 days respectively. Three mutants from the MDS42 strain at day 14 grew as fast or faster than the non-mutant MDS42 strains (see Figure 2), and hence, had wash out times that were significantly faster (greater than or equal to 4000 hours) than the majority of mutants. These were not graphed in order to maintain the same scale as the rest of the sampled time points, but would have appeared at the far right of the histogram. Despite these growth rates, these particular mutants were not maintained in the population, washing out before 21 days.
